# Sex differences in hypertension. Do we need a sex-specific guideline?

**DOI:** 10.3389/fcvm.2022.960336

**Published:** 2022-08-23

**Authors:** Renata Cífková, Larysa Strilchuk

**Affiliations:** ^1^Center for Cardiovascular Prevention, Charles University in Prague, First Faculty of Medicine and Thomayer University Hospital, Prague, Czechia; ^2^Department of Medicine II, Charles University in Prague, First Faculty of Medicine, Prague, Czechia; ^3^Department of Therapy No 1, Medical Diagnostics, Hematology and Transfusiology, Lviv Danylo Halytsky National Medical University, Lviv, Ukraine

**Keywords:** epidemiology of hypertension, cardiovascular risk, white coat hypertension, masked hypertension, polycystic ovary syndrome, contraceptive agents, antihypertensive treatment, large clinical trials in hypertension

## Abstract

Hypertension is the most prevalent cardiovascular disorder and the leading cause of death worldwide in both sexes. The prevalence of hypertension is lower in premenopausal women than in men of the same age, but sharply increases after the menopause, resulting in higher rates in women aged 65 and older. Awareness, treatment, and control of hypertension are better in women. A sex-pooled analysis from 4 community-based cohort studies found increasing cardiovascular risk beginning at lower systolic blood pressure thresholds for women than men. Hormonal changes after the menopause play a substantial role in the pathophysiology of hypertension in postmenopausal women. Female-specific causes of hypertension such as the use of contraceptive agents and assisted reproductive technologies have been identified. Hypertensive disorders in pregnancy are associated with increased risk of maternal, fetal, and neonatal morbidity and mortality, as well as with a greater risk of developing cardiovascular disease later in life. Hypertension-mediated organ damage was found to be more prevalent in women, thus increasing the cardiovascular risk. Sex differences in pharmacokinetics have been observed, but their clinical implications are still a matter of debate. There are currently no sufficient data to support sex-based differences in the efficacy of antihypertensive treatment. Adverse drug reactions are more frequently reported in women. Women are still underrepresented in large clinical trials in hypertension, and not all of them report sex-specific results. Therefore, it is of utmost importance to oblige scientists to include women in clinical trials and to consider sex as a biological variable.

## Introduction

Cardiovascular disease (CVD) has been and continues to be the leading cause of death in women in most developed countries, with coronary heart disease (CHD) being the main contributor in both sexes In Europe, CVD contributes to 49% of total mortality in women, but only to 38% in men (1). The INTERHEART study found that the traditional potentially modifiable risk factors can explain more than 90% of CHD mortality worldwide (2).

Hypertension affects 30–50% of the adult population, making it the most prevalent cardiovascular disorder. At the same time, it is also a strong risk factor for developing CHD, stroke, heart failure, chronic kidney disease, peripheral arterial disease, aortic aneurysm, and atrial fibrillation (3). According to the latest systematic analysis for the Global Burden of Disease Study 2017, hypertension was identified as the leading risk factor responsible for the largest number of total deaths (4).

The objective of this current review is to summarize a comprehensive overview on sex differences in hypertension, from epidemiology through pathophysiology, to treatment.

## Epidemiology of hypertension

### Blood pressure changes over the course of life

There is no difference in blood pressure (BP) in newborns by sex, and the mean BP at birth is around 70/50 mmHg. This is followed by a rather sharp increase in systolic BP over a period of 1–2 years, achieving a BP of 90/55 mmHg at age two. BP increases gradually and continuously in both sexes until around age 12 when the first differences in the sexes become apparent, with boys experiencing a much steeper increase in systolic BP, most likely due to testosterone (5). Males continue to have higher BP values until aged 55 to 60, while the early post-menopausal years are associated with a more pronounced BP increase in women whose BP values then surpass those of males BP and remain higher until the end of life.

### Definition of hypertension

The definition of hypertension has changed over time and the current European guidelines define hypertension as systolic BP ≥ 140 mmHg and/or diastolic BP ≥ 90 mmHg (6). In 2017 the American College of Cardiology/American Heart Association Blood Pressure Guidelines redefined hypertension as systolic BP ≥ 130 mmHg and/or diastolic BP ≥ 80 mmHg (7). However, the studies mentioned in the subsequent paragraph used the 140/90 mmHg definition. None of the definitions of hypertension distinguished between men and women.

### Prevalence

Prevalence rates generally reflect BP values and therefore hypertension is less prevalent in younger women, followed by a steeper increase in prevalence around the menopause. A pooling analysis of 78 population-based studies in adults aged ≥ 20 years reported the overall prevalence of hypertension in women in high-income countries at 23%, while the prevalence in men was 31.6%. In low- and middle-income countries, the overall prevalence of hypertension was almost identical in men (31.7%) and women (31.2%) (8). The US data (NHANES, 2015–2018) showed the prevalence of hypertension in women aged 65–74 years to be 75.7 %, whereas men had a prevalence of 67.5 % (9). Similar results were found in Canada (10) and several developing countries (11). In addition, isolated systolic hypertension is more common in women above the age of 50 (12).

### Awareness

Awareness or previous diagnosis of hypertension is an absolute prerequisite for treatment and control of hypertension. Recent data from the NHANES (2015–2018) survey reported hypertension awareness at 61.2% with higher rates in women of all races/ethnicities (9). Similarly, a systematic analysis by Mills showed higher awareness among women in both high (72.2%) and low- to middle-income countries (44.7%) (8).

### Treatment and control

Treatment and control rates of hypertension are usually better in most population studies in women with better, but still unsatisfactory results in high-income countries. Globally, 43.6% of women with hypertension reported being treated by antihypertensive drugs, but BP control (defined as achieving BP <140/90 mm Hg) was achieved only in 38% (8). The same systematic analysis in men found 30.3% being treated and 35.8% controlled. However, in secondary prevention of CHD, treatment was less frequently initiated in women who also had worse control of their hypertension. While the most recent EUROASPIRE V showed little to no differences in medical treatment between males and females, control of hypertension remained worse in women, which could be due to lower adherence and persistence to antihypertensive medication (13). Additionally, women may have more depression than men. The multi-ethnic study of atherosclerosis (MESA), initiated in six communities in the U.S. in 2000–2002, with follow-up examinations approximately every 2 years, found sex disparities in hypertension control only in the elderly. After adjustment for demographics and co-morbidities, women aged 65–74 years had less controlled hypertension. These sex disparities increased with age and were greatest in the age-group 75+ years (14).

## Blood pressure and cardiovascular risk

Recently published sex-pooled analyses included data from four community-based cohort studies (Framingham Heart Study, Multi-Ethnic Study of Atherosclerosis, Atherosclerosis Risk in Communities Study, and Coronary Artery Risk Development in Young Adults Study) and found increasing CVD risk beginning at lower thresholds of SBP for women than for men (15). In multivariable-adjusted analyses, SBP 100 to 109 mm Hg compared to SBP < 100 mm Hg was associated with incident CVD in women but not in men, in whom increased risk was observed at SBP 130 to 139 mm Hg. This sex-specific difference was found for myocardial infarction (MI), heart failure (HF), and stroke. The MI risk for women with SBP 110 to 119 mm Hg corresponded with the MI risk for men with SBP ≥ 160 mm Hg; the HF risk for women with SBP 110 to 119 mm Hg was comparable to HF risk in men with SBP ranging from 120 to 129 mm Hg. Finally, the risk of stroke in women with SBP 120 to 129 was similar to the risk in men with SBP 140 to 149 mm Hg. Thus, there might be a sex-specific optimal range of SBP, which might be associated with sex differences in vascular anatomy and physiology (e.g., smaller arterial diameter of coronary arteries in women after adjusting for body size) (16). Consequently, sex-specific BP thresholds for initiating drug treatment and BP targets might be defined in the future, provided the results of this observational study are confirmed by a prospective interventional trial.

The International Database on Ambulatory blood pressure in relation to Cardiovascular Outcomes (IDACO) found a steeper increase in risk with 24-h and nighttime BPs in women than in men (17). Nighttime BP was a stronger predictor of outcome than daytime BP. Although the absolute risk is lower in women, the proportion of events potentially preventable by BP lowering was higher in women than in men. A wider implementation of ambulatory BP monitoring in women is desirable.

## White coat hypertension and masked hypertension

The term “white-coat hypertension” refers to the untreated condition in which BP is elevated in the office but is within normal range when measured by ambulatory or home BP monitoring or both (18). On the other hand, “masked hypertension” refers to untreated patients in whom the BP is normal in the office but is elevated when measured outside the office either by home or ambulatory BP monitoring (19).

Women, particularly older or pregnant women, experience white coat hypertension more frequently (20). The ARTEMIS project (Ambulatory Blood Pressure Registry: Telemonitoring of Hypertension and Cardiovascular Risk) found white coat hypertension in 23% of 14,143 patients worldwide. White coat hypertension was more prevalent in elderly, obese women in Europe and Asia (21). Further studies are desperately needed to assess white coat hypertension in women and to determine longitudinal prognosis.

Masked hypertension typically affects older males. The reported worldwide prevalence is 10%, with higher rates in Asia (21). The prevalence of masked hypertension in women increases with obesity and alcohol intake, most likely contributing to their higher CV risk.

## Pathophysiology

**Figure 1:**
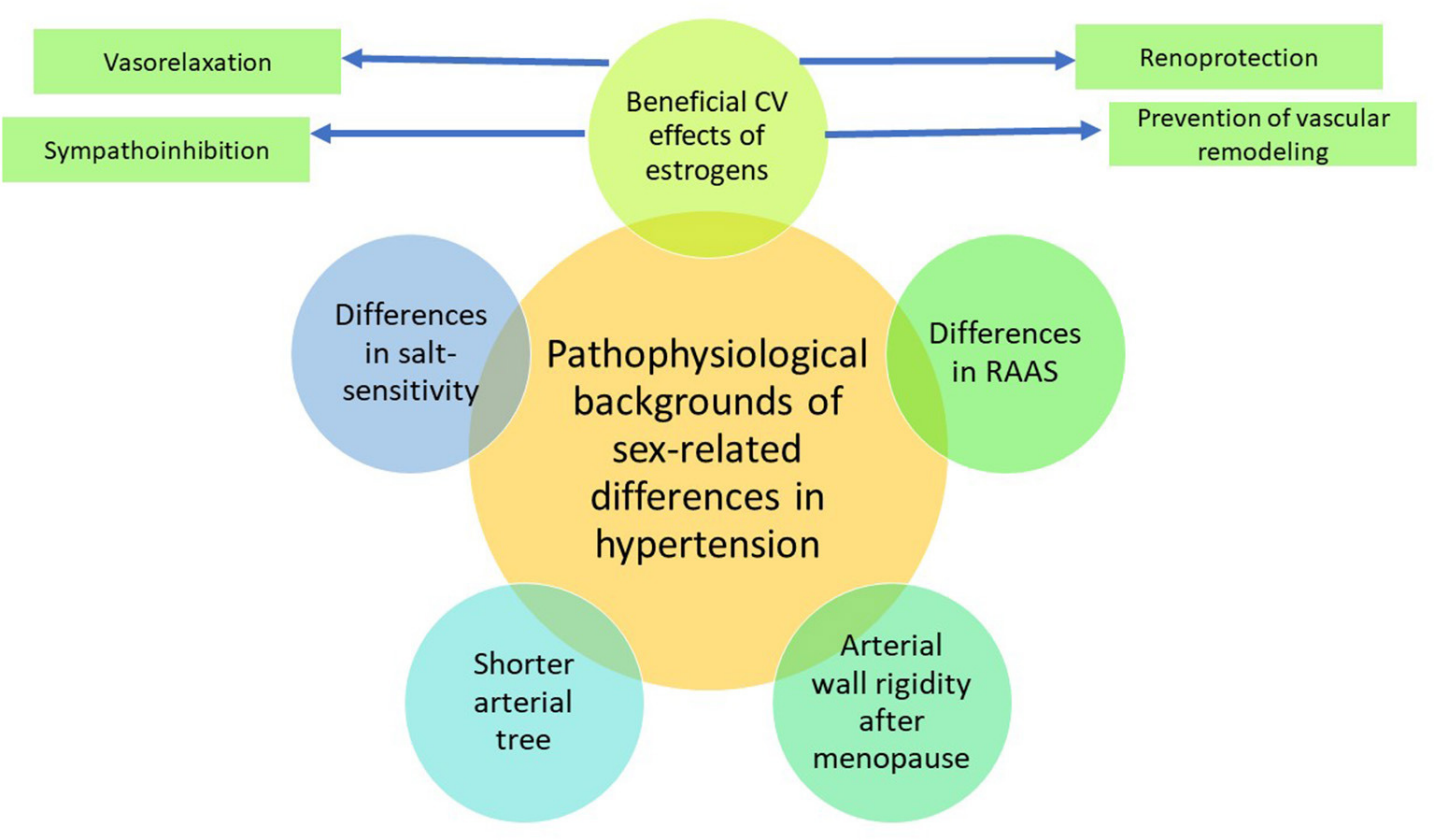
Pathophysiological background of sex-related differences in hypertension. CV, cardiovascular; RAAS, renin-angiotensin-aldosterone system. Sex-related differences in hypertension are mostly explained by the beneficial action of estrogens, promoting vasorelaxation and sympathoinhibition, preventing vascular remodeling, and providing renoprotection. Estrogens also alter the balance between the vasoconstricting and vasodilating arms of the renin-angiotensin-aldosterone system, favoring vasodilation. After menopause, these beneficial effects disappear, and the arterial wall rigidity increases. Apart from that, due to a shorter stature, women have a shorter arterial tree than men, which may lead to amplification of peak systolic blood pressure by the reflected waves.

Although the reasons for differences in BP between males and females are not known, the prevailing proposed hypothesis is that estrogen is responsible for the lower BP in younger women. There are experimental and clinical data suggesting that estrogens have several beneficial cardiovascular effects, such as vasorelaxation, sympathoinhibition, prevention of vascular remodeling and renoprotection (22) (Figure 1). It has been shown that incidence of hypertension was positively associated with testosterone, estradiol, dehydroepiandrosterone and inversely related to sex-hormone binding globulin levels (23).

The increase in systolic BP with age after the menopause is more complex, characterized by the decrease in estradiol and increase in testosterone, resulting in a change of the estrogen-to-androgen ratio. The hormonal changes are followed by endothelial dysfunction, increase in body weight and/or the development of type 2 diabetes.

Estrogens also modulate the renin-angiotensin aldosterone system (RAAS) (24, 25). In addition, sympathetic activation may enhance renin release, thus increasing angiotensin II.

Endothelial dysfunction is also associated with a reduction in nitric oxide and an increase in endothelin, both contributing to salt sensitivity. Angiotensin II and endothelin, and a reduction in nitric oxide and increase in oxidative stress, all contribute to renal vasoconstriction, thus causing hypertension. The increase in arterial stiffness with aging is greater in women, thus inducing greater pulse pressure.

Apart from hormonal changes, there are at least two other hemodynamic differences in women, possibly contributing to the development of hypertension- wave reflections and heart rate (26). Smulyan et al. (27) found a lower pulse pressure in women below age 40 and a higher pulse pressure over 55, which could be explained by early wave reflection due to short stature and higher heart rate in women. The shorter stature in women is associated with a reduced length of the arterial tree, which is thought to be responsible for differences in ventriculovascular coupling. A faster heart rate induces a shorter diastoly, a lower stroke volume, and a lower aortic diastolic BP in all ages in women compared to men (12).

Menopause, due to loss of estrogen, is associated with elastin fragmentation and collagen accumulation in arteries, consequently increasing the rigidity of the arterial wall (28, 29). Therefore, the increase in pulse wave velocity (PWV) is more rapid with aging in women, inducing a more rapid increase in brachial and systolic BP.

In summary, the lower brachial and central diastolic BP in women are consequences of their shorter stature at any age, while the high brachial and central systolic BP and pulse pressure are mostly due to stiffening of conduit arteries after the menopause (26).

In women, the reduced aortic distensibility with aging correlates with increasing levels of follicle-stimulating hormone (30) however, genetic factors may also play a role. Interestingly, telomere length was significantly related to pulse pressure in men, but not in women (31).

Early menopause (age 40–44 years) and relatively early menopause (age 45–49 years) have been shown to be associated with increased risk of CVD. Each year of early menopause is associated with a 3% increased risk of CVD (32).

### Premature ovarian insufficiency

Premature ovarian insufficiency (POI) is the most extreme form of early menopause, defined as natural menopause before the age of 40, affecting approximately 1% of women, with the etiology mostly unknown (33). POI has been shown to be associated with deterioration of lipids, body composition, insulin sensitivity, increase in systolic BP and the risk of metabolic syndrome. Despite the lack of evidence from prospective randomized clinical trials, hormone replacement therapy (HRT) is recommended to be taken until the average age of menopause (34).

### Metabolic peculiarities in women with hypertension

Hypertension is strongly associated with other determinants of cardiometabolic health including body weight, lipid profile, blood glucose, etc. Obesity, dyslipidemia, and impaired glucose tolerance or diabetes mellitus may contribute to the difficulties in normalizing and controlling BP. A cross-sectional study including 6,527 patients from 28 US practices showed that 82% of hypertensive patients had ≥1 additional cardiometabolic risk factor, which was associated with significantly worse BP control compared to participants without these factors (35). About 40% of coronary events in hypertensive men and 68% in hypertensive women are attributable to the occurrence of ≥2 additional metabolic risk factors (36). The cluster of risk factors making up the metabolic syndrome (MS) is associated with an increased relative risk for any CVD (2.88 for men and 2.25 for women, respectively) (37).

As estrogens modulate various aspects of lipid and glucose metabolism, until menopause women have a more favorable metabolic profile than men, whereas after the loss of estrogen signals due to physiological or surgical menopause sex differences gradually diminish (38). According to some authors, menopause may be associated with an increased incidence of hypertension not due to the hormonal changes but due to the increased prevalence of MS in menopausal women (39).

As MS is defined as three or more of its five components (Table 1) (40), it may exist as any of 16 possible combinations of these components and have different pathophysiology, consequences, and treatment options (41).

**Table 1:** Diagnostic criteria for the metabolic syndrome (40).

**Criteria**	**Men**	**Women**
Increased waist circumference	≥94 cm	≥80 cm
Elevated blood pressure	SBP ≥130 mmHg or DBP ≥85 mmHg or antihypertensive drugs	
Elevated fasting glucose	≥5.5 mmol/l or glucose lowering drugs/insulin	
Reduced HDL-C	≥1 mmol/l	<1.3 mmol/l
Elevated triglycerides	≥1.7 mmol/l or lipid lowering drugs	

SBP, systolic blood pressure; DBP, diastolic blood pressure; HDL-C, high-density lipoproteins cholesterol.

In the National Health and Nutrition Examination Survey (NHANES) III (*n* = 33,994), the most prevalent combination of MS components in subjects aged ≤ 65 years included increased triglycerides and decreased HDL-C in both sexes together with elevated BP in men and larger waist circumference in women. All combinations of MS features that were more typical for women included increased waist circumference, whereas the combinations in men were more heterogeneous (41). In Polish nationwide studies WOBASZ and WOBASZ II, abdominal obesity also was the most frequent component of MS in women of all age groups, whereas the male population was mostly characterized by an increased BP (42). Cross-sectional data from 74,531 Western European participants from the Dutch Lifelines Cohort Study confirms that abdominal obesity dominates among MS components in women. In men aged < 60 years, BP was the most common MS component (49.6%), followed by increased triglycerides (24.1%) and decreased HDL-C (22.1%). In women aged < 60 years, abdominal obesity took place in 39.0%, elevated BP in 25.2%, and decreased HDL-C—in 18.5%. In subjects aged ≥60 years, the sex differences diminished (43). So, abdominal obesity may play a more important role in the increase of total cardiometabolic risk in females compared to males.

## Etiology of hypertension

As in men, in 90–95% of female patients with hypertension, no cause is identified. The two main causes of secondary hypertension in women of childbearing age are primary aldosteronism (PA) and renal fibromuscular dysplasia (FMD).

Fibromuscular dysplasia causing renal artery stenosis is more common in women and must be ruled out in all younger women presenting with grades 2 and 3 hypertension, including resistant hypertension, or rapidly developing hypertension. A typical case of renal FMD is a middle-aged white woman with hypertension, usually diagnosed below the age of 30. Fibromuscular dysplasia, by definition, is a non-atherosclerotic arterial disease characterized by abnormal cellular proliferation and distorted architecture of the arterial wall (44). It is primarily a stenotic disease with aneurysm, dissection, and arterial tortuosity frequently presenting in affected patients. Therefore, FMD should always be suspected in dissected renal artery. For most patients with suspected renal artery FMD, CT angiography is the initial imaging method of choice. Alternatively, contrast-enhanced MR angiography should be used when CT angiography is contraindicated. In specialized centers with sufficient expertise in duplex ultrasound for FMD, this may be used as the first diagnostic procedure. A consensus protocol for catheter-based angiography and PTA in patients with renal artery FMD has been developed, recommending assessment of the stenosis severity by measuring a simultaneous unstimulated, translesional pressure gradient (between the distal renal artery and the aorta). The decision whether to perform balloon angioplasty can be made on a pressure gradient threshold of 10% of the mean aortic pressure. Renal artery stenting is generally not indicated in FMD. A recent meta-analysis of six genome-wide association studies found four risk loci for FMD (PHACTR1, LRP1, ATP2B1, and LIMA1) (45).

Primary aldosteronism is the most common form of secondary hypertension in both sexes, requiring specific treatment and being potentially curable. For case detection of PA, the aldosterone to renin ratio (ARR) is recommended as the first screening test (46). Young females were found to have significantly higher ARR than young males (47, 48). False positive ARR in women with hypertension could result in unnecessary suppression tests, followed by adrenal venous sampling. Higher values of aldosterone and renin were found in the luteal phase than in the follicular phase of the menstrual cycle (48–50). Fluctuations in estrogens and progesterone during the menstrual cycle may complicate the interpretation of ARR and aldosterone suppression tests. Therefore, establishing separate, normal ranges of ARR for males and females has been suggested (47, 48). A Japanese study in 2,122 patients with PA who underwent successful adrenal vein sampling found a unilateral subtype of PA occurring more frequently in younger women and decreasing with age (51).

## Female specific causes of hypertension

### Polycystic ovary syndrome

Polycystic ovary syndrome (PCOS) is the most common endocrinopathy in women of reproductive age presenting with hyperandrogenism and chronic oligo/anovulation, affecting up to 15–20% of women. According to the 2003 Rotterdam criteria, PCOS is defined as a combination of any two or all three of the following: hyperandrogenism, polycystic ovarian morphology, and anovulation (52). PCOS women are at high risk for dyslipidemia, glucose intolerance/ type 2 diabetes, and metabolic syndrome including hypertension. In a study from Denmark, more than 30% of women with PCOS had BP ≥ 130/85 mmHg (53). CVD risk was associated with higher BMI, greater waist circumference, higher BP values, higher glucose lipids, and insulin concentrations. Some studies have shown that hyperandrogenism in women with PCOS was associated with the development of hypertension (54, 55).

PCOS women were found to be at increased risk of pregnancy-related complications such as hypertension, preeclampsia, gestational diabetes, and increased neonatal morbidity (56).

A recent meta-analysis confirmed a greater risk of hypertension in PCOS patients only in women of reproductive age, but not in post-menopausal women with a history of PCOS during their reproductive years (57).

### Hypertension associated with the use of contraceptive agents

Studies dating back to the 1990's found some small increases in BP and development of hypertension in about 5% of oral contraceptive (OC) users (58, 59). These older studies have also shown risk of OC and venous thromboembolism and, to a lesser extent, myocardial infarction (particularly in current smokers), and stroke (60). Over the years, the composition of OCs has changed, containing lower doses of estrogen or ethinyl estradiol and a low dose of second or third generation progestins. More recent studies with newer OCs reported conflicting results. Moreover, new hormone delivery systems have been introduced, such as implants, intrauterine systems, and injectables, showing comparable risks to combined OCs (61). Progestin-only pills do not show a significant effect on BP or CVD risk.

Women seeking hormonal contraception should be screened for CV risk factors (hypertension, cigarette smoking, obesity, history of CVD, history of deep vein thrombosis or pulmonary embolism). According to the 2018 ESC/ESH guidelines, oral OCs are not recommended if BP is elevated, and therefore alternative forms of contraception should be offered. Blood pressure control may improve with discontinuation of combined estrogen-progestin OCs (62).

The American College of Obstetricians and Gynecologists (ACOG) is more liberal in its 2019 practice statement, suggesting that in women with hypertension 140–159/ 90–99 mm Hg, combined OC should not be used unless there is no other method available or acceptable to the patient (63). Progestin-only contraceptives (POC) are generally considered safe in women with hypertension, while combined OCs should be prescribed with caution and in non-smoking women with well-controlled and monitored hypertension, younger than 35 years, and showing no evidence of end-organ vascular disease.

### Hypertension and assisted reproductive technologies

Over the past two to three decades, the use of ARTs has been steadily growing. Children born by these techniques comprise 2–5% of births in developed countries. A comprehensive overview of cohort studies from the inception of ART procedures from 1978 to 2016 included 66 longitudinal studies (7,038,029 pregnancies; 203,375 following any ART). Risk ratios for gestational hypertension, preeclampsia, and a combination of both (+ 54% [95% CI, 39–70%]) were increased following any invasive ART independent of gestation (64). The relative risk changes following ART translated into increased absolute numbers of hypertensive complications. A recent meta-analysis confirmed that pregnancies conceived by *in vitro* fertilization (IVF), with or without intracytoplasmic sperm fertilization (ICSI), are at higher risk of being complicated by hypertensive disorders and preeclampsia, compared to spontaneously conceived pregnancies (65).

Moreover, there is growing evidence that ART alters cardiovascular function in children conceived by ART. A Swiss study included 54 apparently healthy adolescents (aged 16.5 ± 2.3 years) conceived with ARTs and compared them with 43 age- and sex-matched controls. The study participants were examined again 5 years later. Flow-mediated dilation (FMD) remained impaired in those conceived with ART, and they also had increased pulse wave velocity (PWV), and intima-media thickness (IMT) compared with the control group. Moreover, 8 of the 52 ART participants, but only one of the 43 control individuals fulfilled ambulatory blood pressure monitoring (ABPM) criteria of hypertension (>130/80 mm Hg and/or >95th percentile (66).

### Hypertensive disorders in pregnancy

Hypertensive disorders in pregnancy complicate 5–10% of pregnancies worldwide, with rates likely to rise because of the pandemic of obesity and the increasing age of pregnant women, particularly in high-income countries and in women with higher education. Hypertension in pregnancy is associated with a substantial increase in maternal, fetal, and neonatal morbidity and mortality.

Hypertensive disorders in pregnancy include (Table 2):

- *Pre-existing hypertension* which, by definition, should be diagnosed before pregnancy or before 20 weeks of gestation. Essential hypertension is most common. It usually persists post-partum. Women with pre-existing hypertension have approximately 25% risk of developing preeclampsia.
- *Gestational hypertension* develops after 20 weeks of gestation and usually resolves within 42 days post-partum
- *Preeclampsia* is a sub-unit of gestational hypertension and, by the current European guidelines, is associated with significant proteinuria (> 0.3 g/24 h or albumin to creatinine ratio ≥30 mg/mmol) (67). The International Society for the Study of Hypertension in Pregnancy (68) introduced another, broader definition of preeclampsia not requiring proteinuria, but including other maternal organ dysfunction:
  - Acute kidney injury (creatinine ≥90 μmol/L; 1 mg/dL)
  - Liver involvement (elevated ALT or AST >40 IU/L) with or without right upper quadrant or epigastric pain)
  - Neurological complications (including eclampsia, altered mental status, blindness, stroke, or more commonly hyperreflexia when accompanied by clonus, severe headaches, and persistent visual scotomata)
  - Hematological complications (thrombocytopenia–platelet count <150,000/μL, disseminated intravascular coagulation, hemolysis)
  - Uteroplacental dysfunction (fetal growth restriction, abnormal umbilical artery Doppler waveform or stillbirth).
- *Pre-Existing Hypertension Superimposed on by Gestational Hypertension With Proteinuria*
- *Antenatally unclassifiable hypertension* refers to cases presenting with hypertension after 20 weeks of gestation with no prior BP values available. Therefore, re-classification is necessary after 42 days post-partum.

**Table 2:** Classification of hypertensive disorders of pregnancy by the 2018 ESC guidelines (67).

**Pre-existing hypertension** •either preceding pregnancy or developing before 20 weeks' gestation; usually persisting for more than 42 days postpartum
**Gestational hypertension** •developing after 20 weeks' gestation; usually resolving within 42 days postpartum **1. Without significant proteinuria** **2. With significant proteinuria** **=** **pre-eclampsia** (protein excretion > 0.3 g/day or ACR ≥ 30 mg/mmol)
**Pre-exising hypertension** **+** **superimposed gestational hypertension with proteinuria** •pre-existing hypertension associated with further BP increase and protein excretion > 3 g/day after 20 weeks' gestation
**Antenatally unclassifiable hypertension** •this term is used when BP is first recorded after 20 weeks of gestation and hypertension is diagnosed; re-assessment is necessary after 42 days post-partum.

**ACR, albumin/creatinine ratio**.

Prevention of preeclampsia by low doses of aspirin (100–150 mg/daily) is recommended to women at high or moderate risk of preeclampsia, starting from week 12 to weeks 36–37.

The following conditions are thought to be associated with a high risk of developing preeclampsia:

- hypertensive disease during a previous pregnancy
- chronic kidney disease
- autoimmune disease such as systemic lupus erythematosus or antiphospholipid syndrome
- type 1 or type 2 diabetes
- chronic hypertension.

Moderate risk of preeclampsia is associated with more than one of the following conditions:

- first pregnancy
- age 40 years or older
- pregnancy interval of more than 10 years
- body mass index (BMI) of 35 kg/m2 or more at first visit
- family history of pre-eclampsia
- multiple pregnancy.

Calcium supplementation (1.5–2 g/day orally) is recommended for prevention of preeclampsia in women with a low calcium intake, which is defined as <600 mg/day, starting at the first antenatal visit.

Management of hypertension in pregnancy includes non-pharmacological (diet, exercise, etc.) and pharmacological treatment, with non-pharmacological methods having only minimal evidence-based data available. Regular exercise might be continued with caution and obese women are recommended to avoid gaining more than 6.8 kg (69). Because of a lack of evidence from large clinical trials, most of the recommendations regarding drug treatment of hypertension in pregnancy are the consensual recommendations of experts. Despite the lack of consensus on the exact definition of severe hypertension, most guidelines consider an SBP ≥170 mmHg or DBP ≥110 mmHg in pregnant women to be an emergency condition, requiring hospitalization (67). Intravenous labetalol and hydralazine, as well as immediate release oral nifedipine are suggested as first-line drugs by the American Society of Obstetricians and Gynecologists (70). On the other hand, hydralazine intravenously is no longer the drug of choice in Europe because of its perinatal adverse effects. It can be used when all other drugs have failed to successfully control BP. Urapidil can also be used, while sodium nitroprusside should only be the last choice, and only used for a short period of time, as prolonged treatment may be associated with an increased risk of fetal cyanide poisoning. In pulmonary edema, nitroglycerine (glyceryl trinitrate) in intravenous infusion seems to be the drug of choice. Immediate release oral nifedipine is still used by many obstetricians, despite possibly inducing a rapid and pronounced BP decrease, followed by tachycardia and subsequent BP increase. The American guidelines suggest using immediate release oral nifedipine as a first line therapy, particularly as a bridge when IV access is not available.

Treatment of mild to moderate hypertension in pregnancy is more complicated and the recommendations of professional societies differ substantially, as the evidence, based on the Cochrane Review, 2018 (71) showed only a significant reduction in risk of developing severe hypertension. A recently published systematic review, network meta- and trial sequential analyses, which included 6,923 women in 72 trials found that labetalol may also decrease proteinuria, preeclampsia, and fetal/newborn death (72). The CHAP study found that a strategy of targeting a BP of <140/90 mmHg was associated with better pregnancy outcomes than a strategy of treating only severe hypertension. There was no increase in the risk of small-for-gestational-age birth weight (73).

### Long-term cardiovascular risk in women with hypertension during pregnancy

There is a substantial and consistent body of evidence showing that hypertensive disorders of pregnancy, particularly with preeclampsia and gestational hypertension, are associated with increased risk of CVD in later life (74). The risk of CVD increases gradually from moderate to severe preeclampsia. Women with preeclampsia have about 3 times (RR 3.13; 95% CI, 2.51–3.89) higher risk of developing hypertension in subsequent years (75, 76). The data of the PREVEND Group suggest that hypertension develops in 20% of women with preeclampsia within 15 years (76). Therefore, regular BP screening is needed, possibly with the implementation of BP self-monitoring, combined with eHealth technologies (77). For women with hypertension in pregnancy, there is also a greater risk of subsequently developing diabetes (78) and dyslipidemia (79).

Lactation has a protective effect on cardiometabolic parameters, such as lower fasting glucose, triglycerides, insulin resistance, higher HDL cholesterol, and lower BP (80–82). Thus, breastfeeding may reduce long term cardiovascular risk and additionally provide protection against breast and ovarian cancers (83, 84). Systematic reviews have shown that lactation may lower CVD risk by reducing BP and preventing hypertension (85, 86). There is inconclusive evidence on the minimal duration of breastfeeding and CVD protection (87).

**Figure 2:**
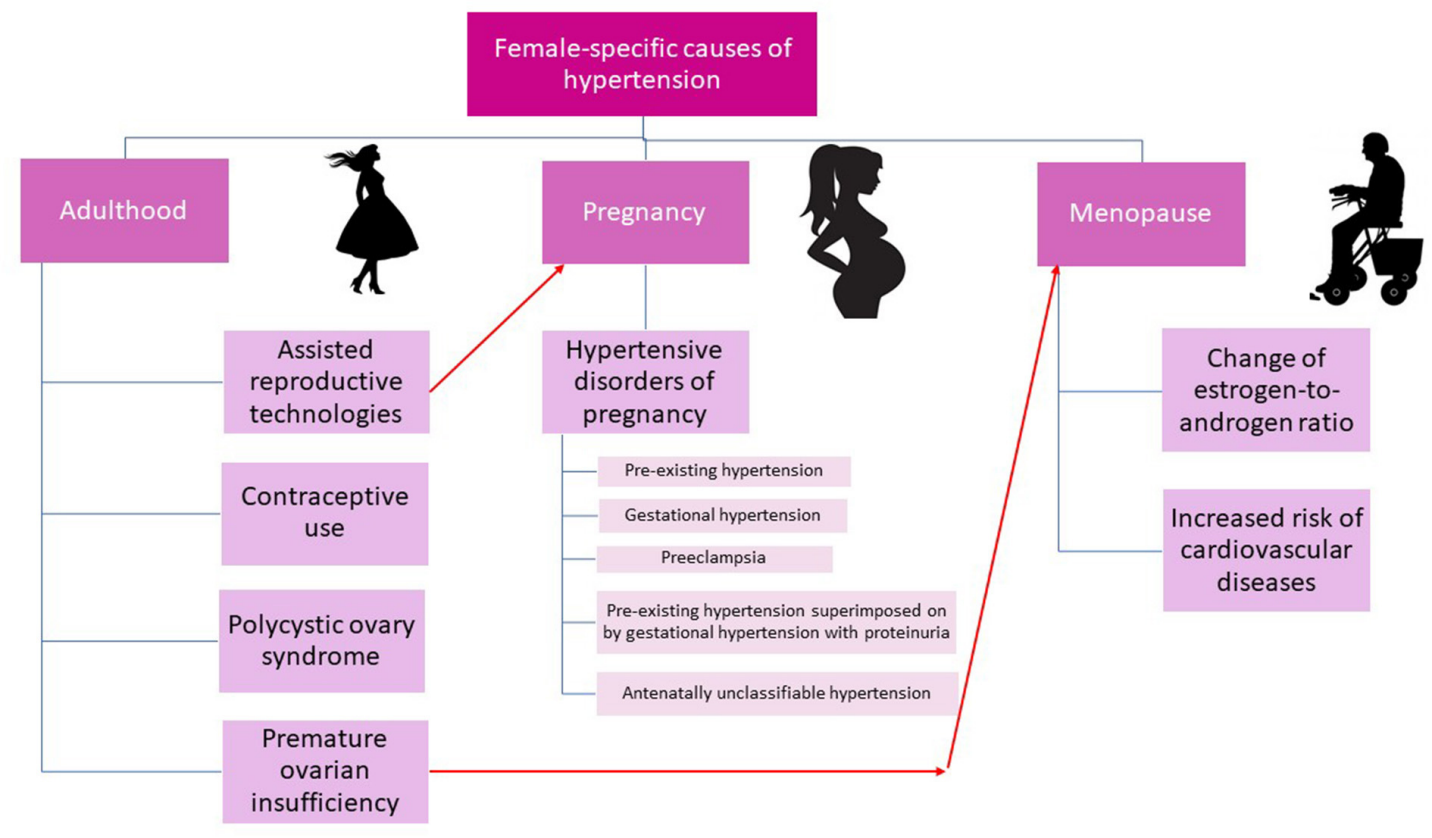
Female-specific causes of hypertension.

Key female-specific causes of hypertension are presented in Figure 2.

## Target organ damage

Hypertension-mediated organ damage (HMOD) reflects structural or functional changes in arteries and organs such as heart, blood vessels, brain, eyes, and kidneys induced by elevated BP, and is considered a marker of pre-clinical or asymptomatic CVD (88). The presence of HMOD is associated with poor prognosis and the CV risk further increases when multiple organs are damaged (89–91).

Several studies have shown that HMOD is more prevalent in women. In the LIFE (Losartan Intervention for Endpoint Reduction in Hypertension Study) echocardiographic sub-study, which included 355 women and 508 men, left ventricular hypertrophy (LVH) was more prevalent in women both at baseline (80 vs. 70%; *p* < 0.01) and at the end of the study (50 vs. 34% *p* < 0.01) (92). The findings were similar in obese and non-obese women, with persistent LVH being associated with higher arterial stiffness. Similarly, the Campania Salute Network project, conducted in southern Italy in 12,329 treated hypertensive patients, found LVH more prevalent in women than in men (43 vs. 32% *p* < 0.001) (93). During follow-up, LVH was newly detected more frequently in women and in obese individuals (94). Women without LVH had a 35% lower risk of major CV events (hospitalization for acute coronary syndromes, heart failure, atrial fibrillation, or CV death), whereas if women had LVH, their risk was similar to that of men. In the Strong Heart study, in middle-aged Native American women LVH was more common than in men (36 vs. 23% *p* < 0.0001) (95).

A dilated left atrium is considered an early sign of hypertensive heart disease and has been shown to be associated with an increased risk of the development of atrial fibrillation, heart failure, and acute coronary syndromes, regardless of the presence of LVH in hypertension (96). The current European hypertension guidelines (6) present a different range of normal values for the left atrium in men (<18.5 mm) and women (<16.5). Several studies have shown that left atrium dilatation was more common in women with hypertension (97, 98). In the LIFE study, left atrium size did not reduce over the 4.8 year's follow-up period.

Arterial stiffness reflects the rigidity of arterial walls and can be assessed non-invasively using pulse wave velocity (PWV). The gold standard for assessing PWV is carotid-femoral PWV using applanation tonometry (99). Before puberty, girls have higher PWV than boys. After puberty, arterial stiffness decreases in women and increases in men, suggesting evidence for the sex hormone effect on vascular stiffness. There is an increase in arterial stiffness with age in both men and women. However, women show a more rapid increase in stiffness after the menopause. Increased arterial stiffness is an increasingly accepted risk factor for CV events and all-cause mortality. Its independent predictive value has been shown in various patient populations, including hypertensives (100–102). Increased arterial stiffness may precede the onset of hypertension and it is also HMOD. In the Framingham Heart Study, increased PWV was found in 63% of controlled hypertensive patients and in 90% of uncontrolled (103). Thus, older women have a greater aortic stiffness and arterial pulsatility than men, which seems to play a role in women's predominance of isolated systolic hypertension, uncontrolled hypertension, heart failure with preserved ejection fraction, and severe aortic stenosis with normal ejection fraction (104).

## Antihypertensive treatment

### Sex differences in pharmacokinetics

Sex differences in pharmacokinetics have been noted in various studies for the last 40 years. Whether these differences have clinical implications is still a matter of debate (105).

There are sex differences in absorption: women have a higher gastric pH, slower gastric emptying, and longer total gastrointestinal transit time. Due to these differences, bioavailability of some drugs might be affected.

Women usually have a lower bodyweight than men while having a higher percentage of body fat. Therefore, lipophilic substances have a higher volume of distribution in women, whereas hydrophilic substances have a higher volume of distribution in men. In addition to that, there is a lower plasma volume in women and lower average organ blood flow. Alpha1-acid-glycoprotein, one of the drug binding proteins, is reduced by estrogens.

Sex differences in liver metabolism and transporters have been reported. Cytochrome (CYP) 3A4 is involved in the metabolism of about 50% of drugs, with women often having higher clearance of drugs metabolized by this enzyme. Glomerular filtration rate (GFR), tubular secretion, and reabsorption are important determinants of excretion. Women have a lower GFR by 10–20% than men (after adjusting for body size).

### Adherence to antihypertensive medication

Adherence to antihypertensive medication is a critical issue in achieving control of hypertension, however, <50% of patients adhere to the medication 1 year after initiation (106). Not all studies report adherence by sex and most of the studies are based on patient self-reports. Some of them use detailed questionnaires and counting pills. Few studies report prescription fill rates. A meta-analysis of 82 studies including 15,517,457 men and 18,537,599 women found no significant differences in adherence between the sexes. These findings were based on self-reported adherence and pharmacy refill records (107). Studies on apparent treatment-resistant hypertension using the highly sensitive technique of therapeutic drug monitoring found that drug adherence was worse in women (108). This is also consistent with the findings of the Italian meta-analysis where lower self-reported adherence was observed in women aged 65 years and older. However, there is a lot of heterogeneity in publications, partly due to different methods used for assessing adherence. Low adherence in elderly women may be associated with higher rates of depression than in their male counterparts, which is known to be a factor in non-adherence (109).

### Efficacy of antihypertensive treatment

According to the current guidelines of the European Society of Cardiology and European Society of Hypertension (2018) and the American Heart Association guidelines (2017), the BP targets and treatment algorithms are the same for both sexes (6, 7).

Data on the better or worse efficacy of some antihypertensive medications in women are fragmentary, inconclusive, and sometimes conflicting. The IDEAL trial showed that indapamide and perindopril reduced systolic BP more in middle-aged women than in men (110). In the VALUE trial, amlodipine-based treatment in women resulted in a larger BP reduction than in men (111). Higher plasma levels of metoprolol and propranolol in women can induce a greater reduction of systolic BP on exertion compared to men (112).

A meta-analysis by Rabi et al. showed that sex-specific data were available only in 9 of 21 included trials (113). Seven of these nine trials indicated that the renin-angiotensin-aldosterone system (RAAS) blockers may be slightly more effective in men than in women, but the difference was usually rather small. As the mean age of all the trial participants was 67.1 years, we may conclude that these trials mostly included postmenopausal women, so future studies including subgroups of women of different ages and reproductive status are warranted.

Another large meta-analysis of 31 randomized trials including 103,268 men and 87,349 women found that achieved BP reductions were comparable for both sexes in every comparison and all BP-lowering regimens included in the analysis provided similar cardiovascular protection (*p* ≥ 0.08 for coronary heart disease, heart failure, cardiovascular death, and total mortality) (114). A borderline difference (*p* = 0.05) was found for stroke: it was shown that women were better protected by diuretics/beta-blockers (BB) or calcium channel blockers (CCB) than by ACEIs, but the authors attributed this finding to chance.

Currently, there are no sufficient data to support sex-based differences in the efficacy of antihypertensive treatment. Therefore, the sex of the patient should not influence drug selection, but the physician must consider the sex-specific adverse drug reactions (ADRs) and specific contraindications for pregnancy (115). Nevertheless, it is still possible that further studies in large populations of hypertensive women may discover optimal sex-specific treatment regimens.

### Safety of antihypertensive treatment

According to the US Food and Drug Administration Adverse Event Reporting System (FAERS), 50 of the 86 most frequently prescribed antihypertensive drugs in the USA had significant sex differences in their ADR profile (116).

A cross-sectional analysis of the Swedish national pharmacovigilance database showed that ADRs associated with ACEIs and their combinations, with angiotensin receptor blockers (ARB) combinations, diuretics, potassium-sparing agents, and dihydropyridine CCBs were reported more often in women (117). The differences seemed to be linked to the dose exposure. On the other hand, aldosterone antagonists more often triggered ADRs in men. There were no significant sex differences in ADRs for ARBs, sulfonamides, and selective BBs.

Women comprised 55.7% of 33,147 ADR-related hospital admissions registered in the PHARMO Database Network (2005–2017) (118). Female thiazide-users had a higher risk of hypokalemia, hyponatremia, and urinary tract infections than men, whereas women taking RAAS blockers had a lower risk of hospitalization due to syncope and collapse than their male counterparts. An increased risk of electrolyte disturbances in females taking diuretics was also found by the Dutch nationwide study (119). According to Rydberg et al. (117), 86% of the reported cases of thiazide-induced hyponatremia occurred in women.

RAAS blockers are known to be teratogenic, which must be taken into account when planning the treatment in women of reproductive age, because some pregnancies are unplanned or not immediately recognized (120). Therefore, RAAS blockers should not be prescribed to women of reproductive age unless they have reliable contraception.

ACEIs induce dry cough twice as often in women. Women are also more prone to CCB-induced peripheral edema (105, 115, 117, 121).

Antihypertensive drugs may have some intraclass variability in ADR types and prevalence, e.g., the number of ADRs induced by CYP 2D6-dependent BBs (metoprolol, carvedilol, nebivolol, and propranolol) was found to be significantly higher in women than in men, whereas the CYP2D6-independent BBs (sotalol, bisoprolol, and atenolol) did not show any sex disparity in their safety profile (122). Regarding ACEIs, the incidence of cough in the SMILE group of studies (*n* = 3,630) was similar between men and women taking zofenopril or ramipril, but it was significantly higher in lisinopril-treated women compared to men (7.2 vs. 2.8%, *p* = 0.025) (123).

Sex differences in drug efficacy and safety are often attributed to differences in body size and composition, but this paradigm seems oversimplified. This is proven by the fact that some studies reveal sex differences after adjustment for these factors. The higher prevalence of ADR in women emphasizes the need for a sex-based selection of drugs, together with close attention and monitoring of side effects. Decreasing ADRs can increase adherence to antihypertensive medication and, thus, improve BP control. We agree with Zucker and Prendergast, who underline the need for sex parity in both preclinical and clinical research from the very beginning of drug development (124).

### Sex differences in prescription of antihypertensive drugs

A systematic review and meta-analysis by Zhao et al. (125) found that women were less likely to be prescribed angiotensin converting-enzyme inhibitors for treatment of hypertension but were more likely to be treated with diuretics. This systematic review and meta-analysis included 43 studies worldwide with more than 2 million participants (28% women) and analyzed sex differences in CV medication prescription in patients at high risk or with established CVD in primary care.

### Large clinical trials in hypertension

Historically, women were less often included in clinical trials because of the potential teratogenic risks of tested drugs, so male physiology and pathophysiology became a standard reference in biomedical sciences (124, 126). The first large clinical trial in hypertension was conducted solely in men, all taken from the post-WWII US veteran population (127, 128). In general, most drugs marketed before the 1990s had only been studied in male animals and men (118). Later the National Institutes of Health (NIH) issued some acts to oblige scientists to include women in clinical trials and consider sex as a biological variable (129).

Subsequent studies included both men and women based on the elevation of diastolic BP as the main entry criterion, independent of age and sex. The first review of randomized controlled trials by MacMahon et al. showed that a reduction of diastolic BP by 5.7 mmHg was associated with a reduction in stroke and coronary heart disease by 40 and 8%, respectively (130). The first reduction of CV risk in women was demonstrated by the Hypertension Detection and Follow-up Program (131, 132) and in three major trials in isolated systolic hypertension in the elderly, the majority being women (133). However, the absolute benefit from treatment was greater in men.

Table 3 shows the selected key hypertension trials and their main sex-related data.

**Table 3:** Sex-specific results of key trials of antihypertensive treatments (in alphabetical order).

**Trial**	**Total *n***	**Proportion of women**	**Sex-specific results**	**Benefits to women or men**	**Reference**
ACCOMPLISH	11,506	39.5%	The results did not reach significance for females	–	(134)
ALLHAT	33,357	47%	Lisinopril was found to be less effective in preventing stroke in women than chlorthalidone and amlodipine	–	(135)
ANBP-2	6,083	51.0%	Enalapril had a beneficial effect compared to a diuretic only in men	–	(136)
HOPE	9,297	26.7%	Equal benefits in the prevention of major CV events for men and women	=	(137)
HOT	18,790	47%	A reduction of diastolic BP <80 mm Hg was associated with a significant reduction of myocardial infarctions in women, but not in men	+	(138)
LIFE	9,193	53.9%	It was more difficult to induce regression of LVH in women (according to the ECG criteria)	–	(139)
VALUE	15,245	42.4%	In women, amlodipine-based treatment was more effective in the prevention of composite cardiac endpoint than valsartan-based	+	(111)

+, results are in favor of women; =, results are neutral; –, results are in favor of men.

A subgroup meta-analysis by Gueyffier et al. of individual patient data from the INDANA (INdividual Data ANalysis of Antihypertensive intervention trials) database showed significant differences in treatment effects between women and men (140). A total of 20,802 women and 19,975 men were drawn from seven clinical trials, with patients recruited between 1972–1990 and treated mainly with beta-blockers and thiazide diuretics. There was a significant reduction in stroke in women, whereas both coronary events and stroke were reduced in men. Similarly, there was another report indicating that antihypertensive treatment was not equally effective in both sexes (141).

The Hypertension Optimal Treatment (HOT) Study included 18,790 hypertensive patients. A reduction of diastolic BP below 80 mmHg was associated with a significant reduction of myocardial infarctions number in women, but not in men (138).

The US Antihypertensive and Lipid-Lowering Treatment to Prevent Heart Attack Trial (ALLHAT) included 33,357 high-risk hypertensive patients and showed a greater BP reduction with amlodipine than with lisinopril which was associated with a reduction in strokes. Further analysis showed lisinopril to be less effective in women and Blacks, compared with chlorthalidone and amlodipine (135, 142).

The Australian Blood Pressure Study 2 (ANBP-2), in mild hypertension, confirmed a beneficial effect of enalapril (compared to a diuretic) only in men (136). In the Losartan Intervention for Endpoint Reduction in Hypertension (LIFE) study, which enrolled patients with hypertension and ECG signs of left ventricular hypertrophy (LVH), it was more difficult to induce regression of LVH in women (139). This finding could explain why women are more susceptible to the development of congestive heart failure (143).

The subgroup interaction analysis of data of the Valsartan Antihypertensive Long-term Use Evaluation (VALUE) trial showed that, in women, amlodipine-based treatment was more effective regarding prevention of composite cardiac endpoint than valsartan-based, whereas in men it was vice versa. Compared to women, in men, valsartan was more effective in cardiac failure prevention (111).

In the Systolic Blood Pressure Intervention Trial (SPRINT) the primary composite outcome in the intensive treatment group was significantly reduced only in men (27 vs. +16%), but there was no statistical interaction between treatment and sex. The latter suggests that, in general, there are no major sex differences in the SPRINT data. We must emphasize that the male and female populations of SPRINT differed according to their smoking exposure, physical activity, and other clinical parameters (144).

The Heart Outcomes Prevention Evaluation (HOPE) trial did not find a significant difference in other ACEI (ramipril) users of both sexes: men had a 24% relative reduction in major CV events, and women had 21% (137). Nordic Diltiazem (NORDIL) and International Verapamil SR Trandolapril Study (INVEST) also found no differences in the outcome reduction between men and women (145, 146).

Avoiding Cardiovascular Events Through Combination Therapy in Patients Living With Systolic Hypertension (ACCOMPLISH) had compared the combinations ACEI + CCB and ACEI + hydrochlorothiazide. ACEI + CCB combination was superior in decreasing CV events and CV death, but the results did not reach significance for females (*p* = 0.06) (134).

To summarize, despite progress, the female population is still underrepresented in clinical trials. We also agree with the EUGenMed Cardiovascular Clinical Study Group, which suggests that sex-related differences should be analyzed not only in a dichotomic way as men and women, but by also considering the whole spectrum of reproductive, menstrual, and contraceptive issues (147).

## Conclusions and future research

Hypertension is a major contributor to CV morbidity and mortality in females. During the reproductive period women are generally protected from CVD events, but after menopause they experience a change in the estrogen-to-androgen ratio and an increase in arterial stiffness resulting in BP increase. Apart from hormonal changes, women have some hemodynamic differences, which may predispose them to high BP.

Women have better awareness, treatment, and control rates of hypertension than men, but their adherence to antihypertensive medication and persistence are lower, which can be partly explained by higher prevalence of depression. Currently, sex-specific BP thresholds for initiating drug treatment and BP targets have not been established, but they might be defined in the future as increasing CV risk in women was found at lower systolic blood pressure thresholds than in men.

Some physiologic and pathologic female-specific conditions are known to induce hypertension, namely, premature ovarian insufficiency, polycystic ovary syndrome, oral contraceptive intake, use of assisted reproductive technologies, and hypertensive disorders in pregnancy. The most common types of secondary hypertension in women include renal artery stenosis caused by fibromuscular dysplasia and primary aldosteronism.

Hypertension-mediated organ damage was found to be more prevalent in women, thus increasing the cardiovascular risk.

Sex differences in pharmacokinetics have been noted. They may be partly explained by differences in gastrointestinal physiology (women have higher gastric pH, slower gastric emptying, and longer total gastrointestinal transit time), and lower body weight, higher percentage of body fat, lower plasma volume, and lower glomerular filtration rate in women.

Sex-related data on the efficacy of antihypertensive medications are contradictory. There are no sufficient data to support different treatments in men and women. Nevertheless, physicians must consider the higher prevalence of adverse drug reactions in females, namely, hypokalemia, hyponatremia, and urinary tract infections (for thiazides), teratogenicity (for RAAS blockers), dry cough (for ACEIs), and peripheral edema (for CCBs).

**Figure 3:**
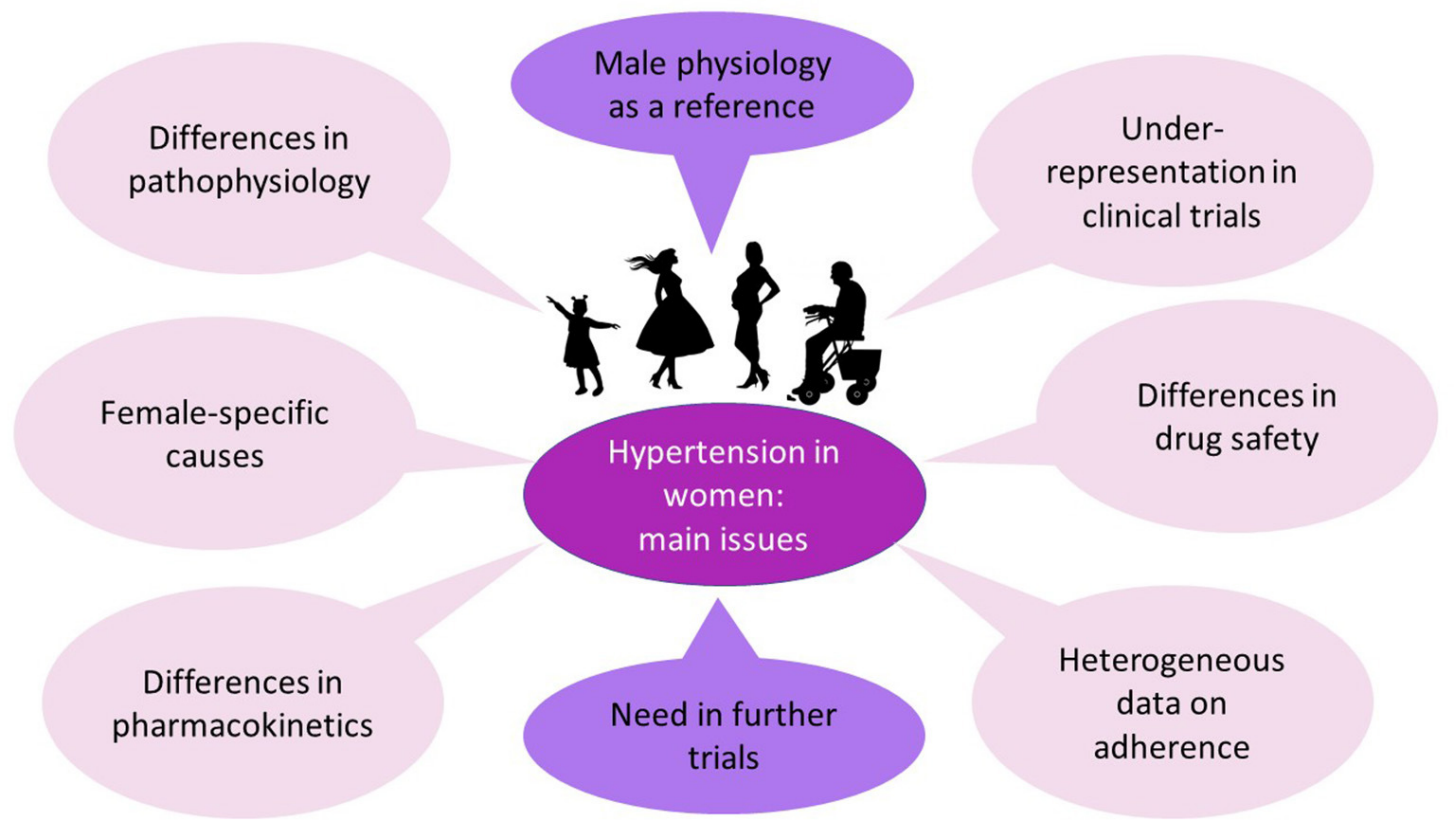
Central Illustration Hypertension in women. Preclinical and clinical trials mainly include male animals and men. Nevertheless, the pathophysiology of hypertension has some sex-specific differences and female-specific causes (these differences and causes are described above). Sex differences in pharmacokinetics and drug safety have also been noted, whereas data on the efficacy of antihypertensive medications are contradictory. Therefore, further trials are needed, and their results may provide background for sex-specific guidelines for hypertension management.

Women are still underrepresented in clinical trials. Moreover, the sex-specific results are usually analyzed in a dichotomic way (men vs. women), whereas there is a need to assess the separate subpopulations of females (oral contraceptive users, women of reproductive age, women in early or timely menopause). Thus, it seems that further research must be sex-specific (Figure 3). This means using female animals for modeling hypertension, testing different drug doses for men and women, and including a wide spectrum of females into clinical trials. In the future, we may discover new data, sufficient to support separate BP targets or different treatment regimens for men and women.

In our opinion, there is still not sufficient data at the present time for sex-specific hypertension guidelines, mostly due to the lack of data on women who were either not included in large clinical trials, or no sex-specific analyses were performed.

## Author contributions

RC conceived and planned the work. RC and LS wrote the manuscript, LS developed the tables and figures. Both authors contributed to the article and approved the submitted version.

## Conflict of interest

The authors declare that the research was conducted in the absence of any commercial or financial relationships that could be construed as a potential conflict of interest.

## Publisher's note

All claims expressed in this article are solely those of the authors and do not necessarily represent those of their affiliated organizations, or those of the publisher, the editors and the reviewers. Any product that may be evaluated in this article, or claim that may be made by its manufacturer, is not guaranteed or endorsed by the publisher.
